# Alternative management of central serous chorioretinopathy using intravitreal metoprolol

**DOI:** 10.1186/s40942-022-00400-5

**Published:** 2022-07-25

**Authors:** Annelise Nicotti Gonçalves, Ingrid U. Scott, Rodrigo Jorge

**Affiliations:** 1 Department of Ophthalmology, Ribeirão Preto Medical School, University of São Paulo, 3900, Bandeirantes Avenue, Ribeirão Preto, SP 14049-900 Brazil; 2 Departments of Ophthalmology and Public Health Sciences, Penn State College of Medicine, 500 University Drive, HU19, Hershey, PA 17033-0850 USA

**Keywords:** B-blockers, Central serous chorioretinopathy, Subretinal fluid, Intravitreal, Retina

## Abstract

**Background:**

Beta-blockers may counteract the effect of catecholamines on central serous chorioretinopathy (CSC) pathology and accelerate the improvement of neurosensory retinal detachment. Oral propranolol has been associated with decreased duration of CSC in some studies. We describe two patients with visually symptomatic chronic CSC (cCSC) treated successfully with intravitreal metoprolol.

**Case presentations:**

After obtaining the patients’ informed consent, two eyes of two 43-year-old men diagnosed with cCSC treated unsuccessfully with oral spirolactone, micropulse laser and intravitreal anti‐vascular endothelial growth factor (anti-VEGF) agents were treated with one off-label intravitreal injection of metoprolol (50 µg/0.05 ml). Baseline (pre-injection) and follow-up examinations (at 1 month post-injection) included best-corrected visual acuity (BCVA), anterior and posterior segment biomicroscopy, fundus autofluorescence, spectral domain optical coherence tomography (Spectralis, Heidelberg), and electroretinogaphy (ERG) according to International Society for Clinical Electrophysiology of Vision (ISCEV) full-field scotopic and photopic standard protocols. ERG results at baseline (pre-injection) and at 1 month post-injection were compared using paired t-tests.

**Results:**

There was no significant difference in any of the ISCEV recommended ERG parameters with respect to a- and b-wave amplitude and implicit time, and oscillatory potentials maximal amplitude. BCVA improved in both patients. Neither patient developed clinical evidence of intraocular inflammation. Subretinal and/or intraretinal fluid had improved in both patients at 1 month after the metoprolol injection.

**Conclusion:**

These preliminary findings suggest that intravitreal metoprolol may be a safe alternative therapy for patients with cCSC.

## Introduction

Central serous chorioretinopathy (CSC) is a common disorder with a multifactorial etiology and unclear pathogenesis that predominantly affects middle-aged men. It is characterized by the presence of serous neurosensory retinal detachment at the posterior pole often associated with focal detachment of the retinal pigment epithelium (RPE), leakage of fluid through the RPE, and hyperpermeability of the choroid [1].

Most cases of CSC resolve spontaneously within 3–4 months, leaving no long-term sequelae except, in some cases, for symptomatic changes in macular function such as metamorphopsia, color discrimination defects, central relative scotoma and contrast sensitivity loss [2]. However, when the subretinal fluid persists, as in the chronic form of the disease, prolonged separation of photoreceptors from the choriocapillaris leads to hypoxic injury to the outer retina and, consequently, visual loss [3]. Persistent subretinal fluid may lead to RPE changes visible on funduscopy and more easily identified on fundus autofluorescence imaging [4]. Chronic CSC may lead to visual acuity impairment due to possible complications such as cystoid macular degeneration, choroidal neovascularization, RPE mottling/atrophy, and disruption of the ellipsoid zone [5].

Although observational management with optimization of modifiable risk factors is an appropriate first-line approach in most cases of CSC, active treatment may be considered when symptoms persist for more than 3 months. There have been a variety of interventions studied for the treatment of CSC, including, but not limited to, verteporfin photodynamic therapy (PDT), laser treatment (argon and micropulse), anti-vascular endothelial growth factor (anti-VEGF) agents, oral beta-blockers, carbonic anhydrase inhibitors, mineralocorticoid receptor antagonist drugs, anti-glucocorticoid medication, anti-Heliobactor pylori treatment, and nutritional supplements (antioxidant supplementation with lutein) [6].

The aforementioned treatment options have variable aftereffects, ranging from laser photocoagulation toxic effects on the RPE and photoreceptors, to anatomic and functional recovery with half-dose PDT [7, 8].

Beta-blockers may counteract the effect of catecholamines on CSC pathology and accelerate the improvement of neurosensory retinal detachment. Oral propranolol has been associated with decreased duration of CSC in some studies [9, 10]. We report two cases of chronic CSC with persistent intraretinal and subretinal fluid treated with intravitreal metoprolol. These cases are part of an ongoing research project with the approval of the local ethics committee.

## Case reports

### Case 1

**Fig. 1:**
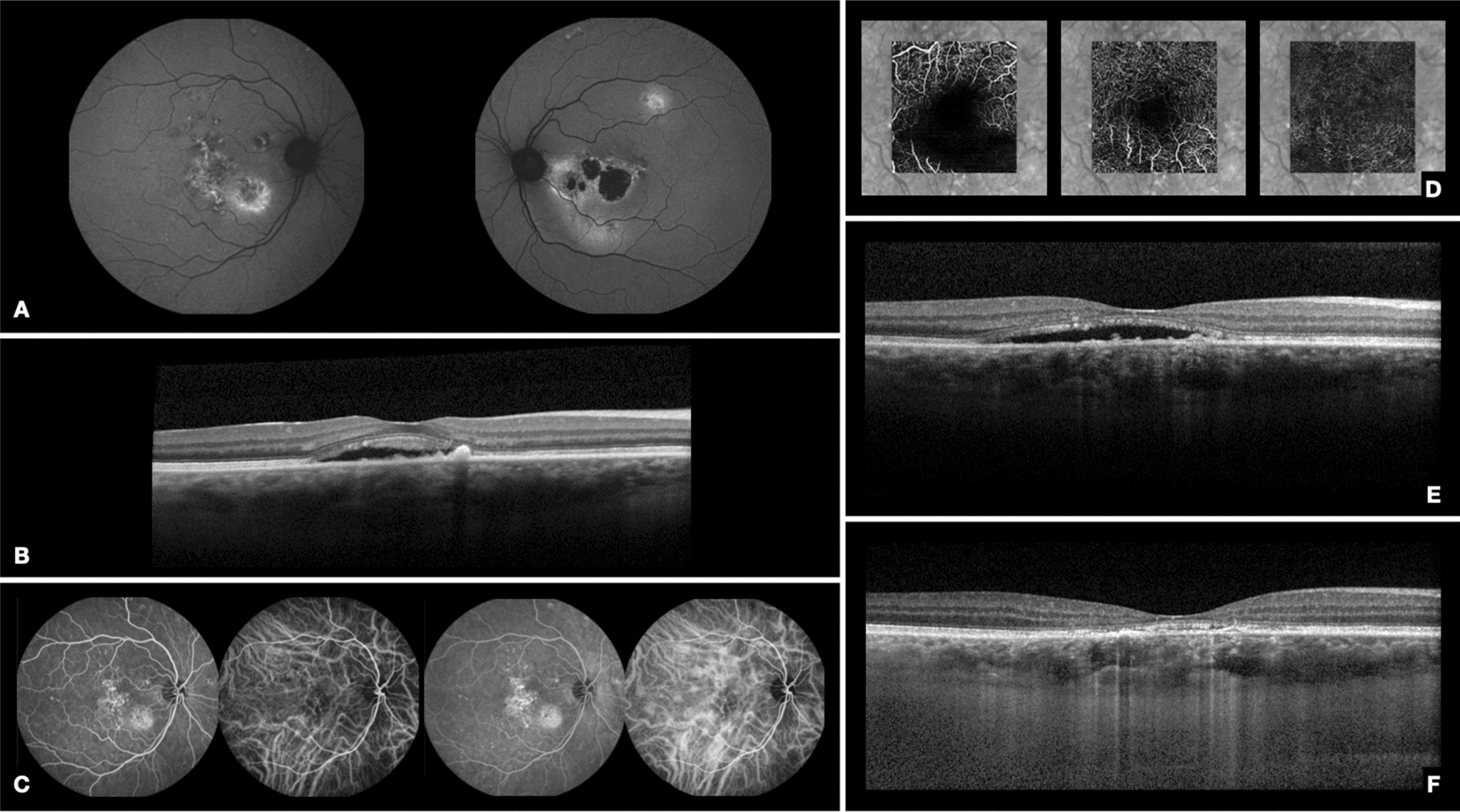
Case 1. **A** Baseline autofluorescence of both eyes. In the OD, there is a mixture of stippled hyper- and hypo-autofluorescence in the posterior pole, including the macula, with extension inferonasally. In the OS, there is a hypo-autofluorescent area in the fovea, with pronounced hyper-autofluorescent regions inferonasal to the fovea. **B** Baseline spectral domain optical coherence tomography (SD-OCT) of the RE shows shaggy photoreceptors and subretinal fluid in the foveal area. **C**–**E** 39 months after baseline, and 1 week before metoprolol injection. **C** Fluorescein and indocyanine green (ICG) angiography of the OD reveal multifocal areas of granular window defect with hyperfluorescent patches. On ICG angiography, there are focal areas of hyperfluorescnce in the initial and middle phases corresponding to areas of RPE changes and choriocapillaris hypermeability. A foveal hyperfluorescent fine-vessel choroidal network was suspected. **D** Optical coherence tomography angiography of the OD shows no signs of choroidal neovascularization. **E** SD-OCT of the OD before intravitreal metoprolol injection demonstrates shaggy photoreceptors, disruption of the ellipsoid zone, and subretinal fluid in the foveal area with RPE irregularity. **F** SD-OCT of the OD 12 weeks after metoprolol injection demonstrates resolution of the subretinal fluid

A 43-year-old man presented with blurry vision in the right eye (OD) for 1 month. He had a several year history of decreased vision in the left eye (OS) treated unsuccessfully with intravitreal anti-VEGF injections (the patient did not know the condition for which he had been treated in the OS). His medical history was positive for systemic arterial hypertension and type 2 diabetes mellitus. On examination, his best-corrected visual acuity (BCVA) was 20/32 in the OD and 20/60 in the OS (ETDRS charts). Slit-lamp examination was unremarkable in both eyes. Fundus examination was notable for RPE mottling in the posterior pole of both eyes, and a tract of RPE atrophy underneath the fovea and extending inferiorly in the OS. Autofluorescence showed focal hypo-autofluorescent dots in the posterior pole of both eyes, and involving the fovea and extending in a descending tract in the OS (Fig. 1A). Spectral domain optical coherence tomography (SD-OCT) demonstrated outer retinal atrophy with disruption of the ellipsoid zone and focal RPE abnormalities in the central macula of both eyes, and subretinal fluid in the fovea of the OD (Fig. 1B). Enhanced depth imaging OCT (EDI-OCT) revealed choroidal thickening in both eyes. Indocyanine green (ICG) angiography demonstrated a possible choroidal neovascular membrane (CNVM) in the fovea of the OD.

Based on the patient’s history and examination findings, a diagnosis of chronic CSC in both eyes with possible CNVM in the OD was made, and the patient was treated in the OD with intravitreal ranibizumab.

After the first three monthly intravitreal ranibizumab injections, the patient’s BCVA in the OD had worsened to 20/60 and SD-OCT confirmed increased subretinal fluid. After three additional monthly intravitreal ranibizumab injections, there was a reduction of subretinal fluid although the BCVA remained 20/60. Over the subsequent 12 months, the patient was treated with 4 more intravitreal injections of anti-VEGF (three of ranibizumab and one of aflibercept); during this time, the subretinal fluid remained stable and the BCVA remained 20/60. However, 1 month after the tenth intravitreal anti-VEGF injection (nine of ranibizumab and one of aflibercept), the patient presented with stable BCVA of 20/60, but with worsening of the subretinal fluid. Until that time, the patient received intravitreal anti-VEGF treatment due to a possible CNVM, but he was not showing a good response. Because of worsening subretinal fluid despite anti-VEGF treatment, micropulse laser was indicated. We opted for micropulse treatment, because PDT is not available in the public health system in Brazil and we were trying available therapies that could contribute to subretinal fluid absorption. The laser was performed using a 810 µm-micropulse laser with a duty cycle of 15%, spot size of 125 microns, and power of 850 mW; 676 spots were delivered to the patient’s OD. Twelve weeks after the laser treatment, the patient’s BCVA remained 20/60 but the subretinal fluid worsened. Oral treatment with spironolactone 25 mg bid was initiated and three additional ranibizumab injections were administered with no improvement in BCVA. Micropulse treatment was repeated 1 month after the last injection (8 months after the first micropulse session) and was followed by an initial small reduction of the subretinal fluid, which then worsened after a few weeks. Two additional ranibizumab injections were administered 20 weeks after the second micropulse session, but did not result in an improvement in BCVA or subretinal fluid.

Thirty-nine months after the patient’s initial visit (3 months after the last injection and 10 months after the second micropulse laser session), BCVA in the OD remained 20/60, and there was persistent subretinal fluid. Fluorescein angiography revealed multifocal areas of granular window defect with hyperfluorescent patches in the macula of both eyes. A foveal hyperfluorescent fine-vessel choroidal network was suspected on ICG angiography of the OD (Fig. 1C). At that time, OCT angiography (OCT-A) became available in our Retina Service and was performed, and showed no evidence of CNVM (Fig. 1D). SD-OCT of the OD demonstrated outer retinal and RPE changes with subretinal fluid (Fig. 1E). Due to failure of the anti-VEGF injections, oral spironolactone, and microplulse laser, off-label use of intravitreal metoprolol (50 µg/0.05 ml) was proposed as a treatment alternative for the patient’s OD. After obtaining the patient’s informed consent, multifocal electroretinography (ERG) was performed and an intravitreal metoprolol injection was then administered without complication.

Four weeks after intravitreal metoprolol injection in the OD, BCVA improved to 20/40 and partial reduction of the subretinal fluid was observed on SD-OCT; CST decreased from 238 to 191 μm, and choroidal thickness from 441 to 410 μm. Twelve weeks after intravitreal metoprolol injection, the patient’s BCVA remained 20/40, and there was minimal subretinal fluid on SD-OCT (Fig. 1F), with a CST of 162 μm and choroid thickness of 403 μm. There were no ERG changes one month after the intravitreal injection of metoprolol.

### Case 2

A 43-year-old man presented with slowly progressive visual acuity loss in the OS for 2 years. He denied any systemic or ocular medical history. On examination, he had a BCVA of 20/20 in the OD and 20/25 in the OS (ETDRS charts). Slit-lamp examination was unremarkable. Fundus examination demonstrated RPE mottling in the posterior pole of both eyes, and a tract of RPE atrophy extending inferiorly from the fovea in the OS.

**Fig. 2:**
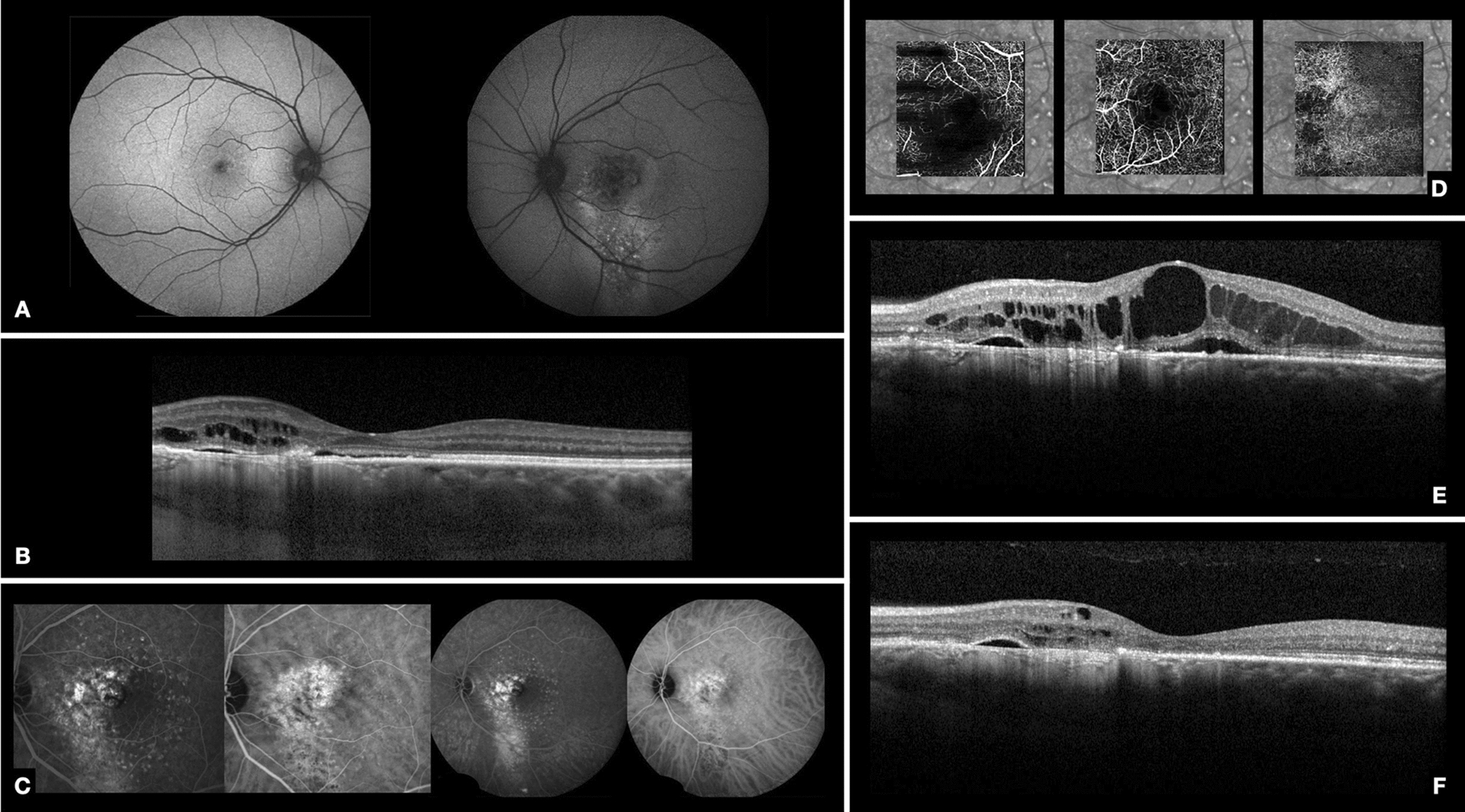
Case 2. **A** Baseline autofluorescence of both eyes demonstrates no significant RPE changes in the OD and, in the left eye, shows focal hypo-autofluorescent dots in the posterior pole, including the macula, and forming a descending tract. **B** Baseline spectral domain optical coherence tomography (SD-OCT) of the OS reveals foveal subretinal fluid. In the nasal macular region, there is outer retina and RPE atrophy, associated with intraretinal and subretinal fluid. **C**–**E** 31 months after baseline, and 1 week before metoprolol injection. **C** Fluorescein angiography reveals transmission hyperfluorescence in the areas corresponding to the RPE changes on autofluorescence. There are also small hyperfluorescent dots surrounding the foveal area, which corresponded to short-pulse laser burns. OS Indocyanine green angiography demonstrates areas of hyperfluorescence secondary to RPE changes and choriocapillaris hyperpermeability in the early and late phases. **D** Optical coherence tomography angiography of the OS shows no signs of choroidal neovascularization. **E** Spectral domain optical coherence tomography (SD-OCT) of the OS before intravitreal metoprolol demonstrates outer retinal atrophy and RPE defects in the foveal area associated with chronic cystoid macular edema. **F** SD-OCT of the OS five weeks after metoprolol injection shows a significant reduction of the intraretinal fluid

The patient’s history and examination findings were suggestive of chronic CSC. Fundus autofluorescence was unremarkable in the OD and, in the OS, demonstrated hypo-autofluorescent confluent dots mixed with stippled autofluorescence in the foveal area, surrounded by a hyper-autofluorescent halo. These changes extended inferiorly from the foveal region to beyond the inferotemporal vascular arcade, creating a descending tract of hyper-autofluorescence in the OS (Fig. 2A). SD-OCT demonstrated minimal RPE irregularity in the OD and, in the OS, there was outer retinal atrophy and RPE defects primarily in the nasal foveal area associated with cystoid macular edema. There was also a hyperreflective change at the level of the RPE/photoreceptors that raised suspicion for a secondary CNVM (Fig. 2B). There was choroidal thickening (516 μm).

The patient’s OS was treated with an intravitreal aflibercept injection, followed 10 days later by 631 spots of 810 µm-micropulse laser with a duty cycle of 15%, spot size of 125 microns, and power of 840 mW. Nine weeks after the laser treatment, BCVA in the OS remained 20/25 and there was partial improvement of the intraretinal fluid. The patient was lost to follow-up and, 1 year after treatment, he returned with a BCVA of 20/125 and cystoid macular edema in his OS. Short-pulse laser was performed (Alcon Purepoint^®^ green diode laser, 100 spots, spot size of 100 microns, and power of 175 mW) and oral spironolactone 25 mg bid was initiated. Eight months later, the patient’s BCVA in the OS was 20/200 and there was persistent intraretinal fluid in the macula of the OS. Two intravitreal injections of ranibizumab were administered, but the BCVA declined to counting fingers at 3 m and SD-OCT showed increased intraretinal fluid, as well as subretinal fluid, in the macula.

Thirty-one months after the initial visit, and six months after the last ranibizumab injection, BCVA was 20/20 in the OD and counting fingers at 3 m in the OS. Autofluorescence was similar to baseline, showing focal hypo-autofluorescent dots in the posterior pole, including in the foveal area and extending in a descending tract, in the OS. Fluorescein angiography showed transmission hyperfluorescence in the areas corresponding to the RPE changes on autofluorescence. Indocyanine green angiography demonstrated areas of hyperfluorescence secondary to RPE changes and choriocapillaris hyperpermeability in the early and late phases in the OS (Fig. 2C). SD-OCT demonstrated persistent intraretinal fluid with a CST of 407 μm (Fig. 2E). OCT-A confirmed the absence of an associated CNVM (Fig. 2D).

Due to the failure of previous intravitreal anti-VEGF pharmacotherapy and laser therapy to the patient’s OS, off-label use of intravitreal metoprolol (50 µg/0.05 ml) was proposed as a treatment alternative. After obtaining the patient’s informed consent, multifocal ERG was performed and an intravitreal metoprolol injection was then administered to the OS without complication. Five weeks after intravitreal metoprolol injection, BCVA in the OS had improved to 20/160 and there was significant reduction of the intraretinal fluid on SD-OCT, with a CST of 186 μm (Fig. 2F). There were no ERG changes one month after the intravitreal injection of metoprolol.

## Discussion

Several different treatment strategies, including PDT using verteporfin, rifampicin, methotrexate, mineralocorticoid antagonists and various laser modalities, have been reported to be effective for chronic CSC. Researchers have reviewed these treatment modalities and concluded that half-dose (or half-fluence) PDT is the most promising treatment for chronic CSC, as recently reviewed by van Rijssen et al. [11] and confirmed by the PLACE study, the largest, prospective multicenter randomized controlled treatment trial for chronic CSC conducted to date [12].

Photodynamic therapy with verteporfin can be used in CSC with focal or diffuse RPE leakage involving the central macula, but carries the risks of transient reduction in macular function, choroidal non-perfusion, foveal injury, RPE rip, CNVM development and progressive RPE atrophy. To minimize these complications, reduced fluence, reduced medication dosage, and reduced laser activation time were studied, and half-dose PDT was reported to be the safest and most efficient of these treatment options for chronic CSC [13]. The laser machine for PDT with verteporfin is no longer registered by the Brazilian Health Agency and this treatment is no longer available in the Brazilian public health system. In addition to the difficulty of access to PDT treatment in some countries, the high cost, including expenses with the drug and its infusion, also limits its use [14].

Since the two patients described in this report had already failed several oral, intravitreal and laser treatments, the authors offered the patients off-label intravitreal treatment with a ß1-selective adrenergic blocker: metoprolol. Oral and intravenous formulations of metoprolol are used for the treatment of systemic hypertension, and topical metoprolol has been used for ocular hypertension and glaucoma treatment. Metoprolol tartrate has proven to be nontoxic to RPE cells, and intraperitoneal and oral metoprolol have been reported to be neuroprotective in a rat model of retinal degeneration [15]. Its intravitreal use has been reported for circumscribed choroidal hemangioma [16]. Further, our group published an experimental study in which no evidence of retinal toxicity was found following administration of intravitreal metoprolol at doses of 50 and 100 µg in rabbit models, through analysis of clinical observation, electroretinography and histological evaluation [17]. The rationale for adrenergic blockade to treat CSC is that patients with CSC have been demonstrated to have markedly elevated plasma epinephrine, with epinephrine levels correlating with central macular thickness, macular edema, and visual acuity [18]. In vitro porcine RPE cells underwent apoptosis following exposure to elevated levels of epinephrine [19]. Excess epinephrine may affect the retina through RPE beta-adrenergic receptors and changes in cyclic adenosine monophosphate concentration; the latter affects RPE electrical activity [20]. Beta-adrenergic antagonists seem to prevent changes in RPE activity and epinephrine-induced apoptosis, which compromise RPE integrity and contribute to CSC. In fact, another ß-blocker, propranolol, has been used for CSC as oral monotherapy in cases of persistent leakage and has been associated with improvement in BCVA, central macular thickness, and leakage on fluorescein angiography [9, 10]. Kianersi and Fesharaki conducted a double-blind randomized controlled clinical trial with 60 CSC patients divided into two groups: placebo versus propranolol. They observed that duration of disease and need for laser therapy were decreased by the use of propranolol, but there was no difference between the treatment groups with respect to final vision [10]. Tatham and Macfarlane published a series of two patients treated with oral propranolol for CSC. One of the patients demonstrated recovery (visual acuity improvement and subretinal fluid reabsorption), recurrence (visual acuity deterioration and recurrent of neurosensory detachment), and subsequent re-recovery (visual acuity was 6/6 and the retina was once again attached), which coincided with the commencement, cessation, and retreatment with oral propranolol [9]. Unfortunately, there is no aqueous solution of propranolol in the Brazilian market and, for this reason, the aqueous solution of metoprolol was chosen for the treatment of the patients reported in the current series. The dosage of 50 µg was selected based on the concentration of the commercially available solution of the drug, and to achieve adequate volume for intravitreal injection of 0.05 ml. This dosage is 4.000 times lower than the usual oral dosage advised for systemic arterial hypertension treatment; thus, side effects associated with systemic b-blockers would be expected to be minimized.

The intravitreal use of metoprolol does not seem to cause retinal toxicity in the short term. There were no ERG changes at 1 month after the intravitreal injection of metoprolol in both patients with chronic CSC, as was the case in a patient treated with intravitreal metoprolol for choroidal hemangioma reported by Jorge et al. [16]. In addition, there was significant reabsorption of subretinal fluid and reduction in CST in both patients in the current series. Notably, the fluid reabsorption had occurred by one month after metoprolol injection despite the fact that the patients had undergone multiple other CSC treatments for months before being treated with metoprolol. The temporal correlation between metoprolol treatment and fluid absorption provides further evidence for the efficacy of ß-blocker action on the anatomical improvement in patients with CSC. The improvement of CST was accompanied by choroidal thickness reduction in both patients. Since there is evidence that the sympathetic nervous system affects choroidal blood flow, and that subcutaneous propranolol may increase choroidal thickness in rats [21], the reduction in choroidal thickness may be a coincidental finding, or perhaps the effect in CSC patients is different and the reduction in choroidal thickness may contribute to a reduction in subretinal and intraretinal fluid. Finally, paralleling fluid and CST improvement, there was also BCVA improvement in both patients following treatment with intravitreal metoprolol. This improvement reinforces the safety of metoprolol, and is consistent with the demonstrated ERG stability.

Despite the above-mentioned results that suggest intravitreal metoprolol safety for CSC treatment, it is important to mention that two cases represent limited information and a larger number of patients must be studied to confirm our preliminary findings.

## Conclusion

The current report is the first to report anatomical and functional results with intravitreal ß-blocker treatment in patients with chronic CSC. The reabsorption of intraretinal and subretinal fluid after 1 month of follow-up in patients who had already failed several oral, intravitreal and laser treatments points to intravitreal metoprolol as an effective alternative therapy for chronic CSC in the two patients reported herein. However, the therapy may not be equally effective for all patients, and more than one injection may be necessary. Although ß-blockers may counter the effect of catecholamines on CSC pathology, the potential risks of long-term retinal toxicity and complications from repeated intravitreal injections, and the need for more information on the risk:benefit ratio associated with intravitreal metoprolol treatment, warrant further research with a larger number of patients and longer follow-up.

## Data Availability

Data sharing is not applicable to this article as no datasets were generated or analyzed during the current study.
